# SARS-CoV-2 Nsp15 suppresses type I interferon production by inhibiting IRF3 phosphorylation and nuclear translocation

**DOI:** 10.1016/j.isci.2023.107705

**Published:** 2023-08-23

**Authors:** Dianqi Zhang, Likai Ji, Xu Chen, Yumin He, Yijie Sun, Li Ji, Tiancheng Zhang, Quan Shen, Xiaochun Wang, Yan Wang, Shixing Yang, Wen Zhang, Chenglin Zhou

**Affiliations:** 1 Clinical Laboratory Center, The Affiliated Taizhou People’s Hospital of Nanjing Medical University, Taizhou 225300, China; 2 Jiangsu Key Laboratory of Medical Science and Laboratory Medicine, School of Medicine, Jiangsu University, Zhenjiang, Jiangsu 212013, China; 3 Department of Clinical Laboratory, The Affiliated Yixing Hospital of Jiangsu University, Yixing, Jiangsu 214221, China; 4 Department of Laboratory Medicine and Pathology, Jiangsu Provincial Corps Hospital of Chinese People’s Armed Police Force, Yangzhou, Jiangsu 225003, China; 5 Medical Research Center, Northern Jiangsu People’s Hospital, Yangzhou, Jiangsu 225001, China

**Keywords:** Immunology, Virology, Cell biology

## Abstract

Severe acute respiratory syndrome coronavirus 2 (SARS-CoV-2), which causes 2019 coronavirus disease (COVID-19), poses a significant threat to global public health security. Like other coronaviruses, SARS-CoV-2 has developed various strategies to inhibit the production of interferon (IFN). Here, we have discovered that SARS-CoV-2 Nsp15 obviously reduces the expression of *IFN-β* and IFN-stimulated genes (*ISG56*, *CXCL10*), and also inhibits IRF3 phosphorylation and nuclear translocation by antagonizing the RLR-mediated antiviral signaling pathway. Mechanically, we found that the poly-U-specific endonuclease domain (EndoU) of Nsp15 directly associates with the kinase domain (KD) of TBK1 to interfere TBK1 interacting with IRF3 and the flowing TBK1-mediated IRF3 phosphorylation. Furthermore, Nsp15 also prevented nuclear translocation of phosphorylated IRF3 via binding to the nuclear import adaptor karyopherin α1 (KPNA1) and promoting it autophagy-dependent degradation. These findings collectively reveal a novel mechanism by which Nsp15 antagonizes host’s innate immune response.

## Highlights

• SARS-CoV-2 Nsp15 inhibited RLR-mediated interferon production
• Nsp15 interferes with the interaction between TBK1 and IRF3 by competing with TBK1
• Nsp15 reduced IRF3 nuclear translocation by degrading KPNA1

## Graphical abstract

**Figure:**
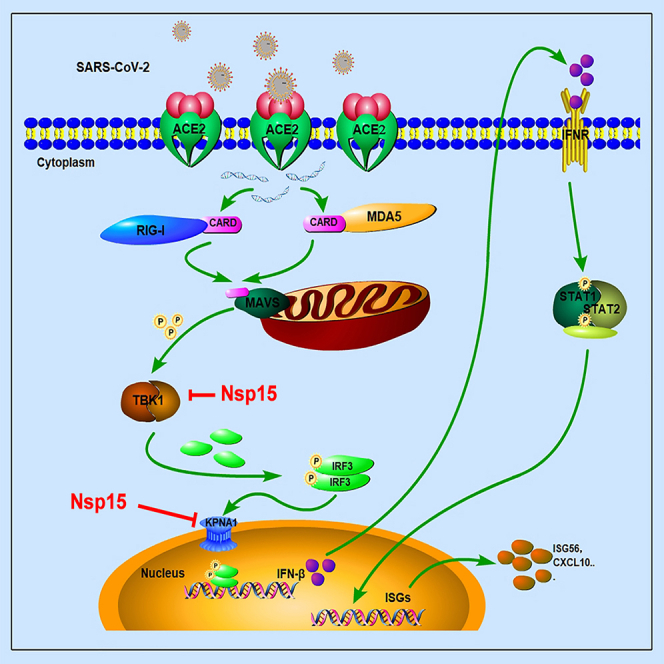

## Introduction

The severe acute respiratory syndrome coronavirus 2 (SARS-CoV-2) has caused the outbreak of coronavirus disease 2019 (COVID-19) worldwide, posing a significant threat to human life.^1^ SARS-CoV-2 is a single positive-stranded RNA virus in the family *Coronaviridae*, genus *Betacoronavirus*. Its viral genome primarily encodes two large open reading frames (ORFs), ORF1a and ORF1b, which are then translated into two large replicase polyprotein precursors (pp1a and pp1b). The polyproteins are cleaved into 16 nonstructural proteins by papain-like proteinase (Nsp3) and 3C-like proteinase (3CLpro), also known as Nsp5.^2^ Aside from these nonstructural proteins, sub-genomic RNA of SARS-CoV-2 encodes four structural proteins (spike envelope, membrane, nucleocapsid) and several accessory proteins.^3^ Early clinical features of COVID-19 patients were mild or even asymptomatic, but the peak in SARS-CoV-2 RNA concentrations occurred earlier (around day 4) compared to SARS (around day 7–10).^4^^,^^5^ Previous studies have also shown that the suppression of antiviral immune responses in cells or animals following SARS-CoV-2 infection.^6^ Gaining a clearer understanding of the molecular-level mechanisms of virus-host interactions is crucial for comprehending COVID-19 pathogenesis and transmission.

The wide spread of SARS-CoV-2 is largely attributed to specific viral pathogenesis and the ability to avoidance of immune surveillance.^7^^,^^8^ The host’s innate immune system acts as the first line of defense against viral infections. The type I interferon (IFN) system plays a crucial role in the innate immune response. Upon infection, pattern recognition receptors (PRRs) recognize viral pathogen-associated molecular patterns (PAMPs) to activate the innate immune system. Retinoic-inducible gene-I (RIG-I)-like receptors (RLRs), including RIG-I, melanoma differentiation-associated gene-5 (MDA5) and the Laboratory of Genetics and Physiology 2 (LGP2), serve as critical cytosolic RNA sensors. After recognition of cognate ligands, such as double-strand RNA (dsRNA), triggers RIG-I to expose the caspase activation and recruitment domain (CARD), and then interacts with the CARD domain of the adapter mitochondrial antiviral signaling protein (MAVS, also known as IPS-1/VISA). Activation of MAVS recruits and activates tank-binding kinase 1 (TBK1) and the inhibitor of κ-B kinase ε (IKKε), which promote the phosphorylation of interferon regulatory factor 3 (IRF3). Phosphorylated IRF3 then translocate to the nucleus to stimulate the production of Type I IFN.^9^^,^^10^^,^^11^ IFN is a class of cytokines that plays an antagonistic role in viral replication and transmission by inducing the expression of IFN-stimulated genes (ISGs).

Human coronaviruses have developed various strategies to evade innate immunity.^12^ Recently, studies have shown that some proteins encoded by SARS-CoV-2 can antagonize the production of IFNs. SARS-CoV-2 ORF9b antagonized the RIG-I/MAVS antiviral immune response by interrupting K63-linked polyubiquitination of the IFN signaling modulator NEMO.^13^ Nsp12 attenuated type I IFN response by inhibiting IRF3 nuclear translocation.^14^ SARS-CoV-2 Nsp5 and N proteins could interfere with the formation of antiviral stress granules and prevent the phosphorylation of TBK1 and IRF3, thereby blocking IRF3’s translocation into the nucleus.^15^ Nsp5 also has two distinct mechanisms to evade the innate immune response by targeting RIG-I and MAVS.^16^ Furthermore, using the dual-luciferase reporter system, Pei-Yong Shi et al. identified Nsp6, Nsp13, and ORF6 as IFN production antagonists, Chun-Kit Yuen et al. identified Nsp13, Nsp14, Nsp15, and ORF6 as potent IFN antagonists, and Maya Shemesh et al. identified Nsp1, Nsp5, Nsp6, Nsp15, ORF6, and ORF7b as IFN-β production blockers.^17^^,^^18^^,^^19^

Coronavirus Nsp15 is a conserved uridine-specific endonuclease. Each Nsp15 protomer consists of three major domains: an N-terminal domain, a variable middle domain, and a C-terminal endonuclease (endoU) domain.^20^ As an integral component of the coronavirus replication-transcriptase complex (RTC), Nsp15 plays a crucial role in the RTC-mediated viral immune escape process.^21^^,^^22^ Negative-sense (PUN) RNAs containing 5′-Poly U, which are viral pathogen-associated molecular patterns (PAMPs) can be recognized by RLRs to activate the IFN response. However, the endonuclease activity of coronavirus Nsp15 cleave PolyU sequences from PUN RNAs, allowing the virus to evade the innate immune system.^23^ Additionally, it has been discovered that SARS-CoV-2 Nsp15 also inhibits *de novo* autophagy induction.^24^ Previous studies have shown that SARS-CoV-2 Nsp15 could antagonize the host’s innate immune response,^17^^,^^19^ but the specific mechanism behind this remains to be determined.

Here, we report that Nsp15 suppresses IFN production in two different ways: (1) Nsp15 weakened the interaction between TBK1 and IRF3 by competitively combining with TBK1, thereby reducing IRF3 phosphorylation; (2) Nsp15 inhibited nuclear translocation of phosphorylated IRF3 via binding and reducing karyopherin α1 (KPNA1) protein expression. To our knowledge, this is the first time to clarify the mechanism of Nsp15 antagonizing the innate immune pathway in detail. This study will provide new insight into molecular mechanism of SARS-CoV-2 escaping host’s antiviral innate immunity.

## Results

### Screening and testifying of potential interferon antagonists encoded by SARS-CoV-2

**Figure 1:**
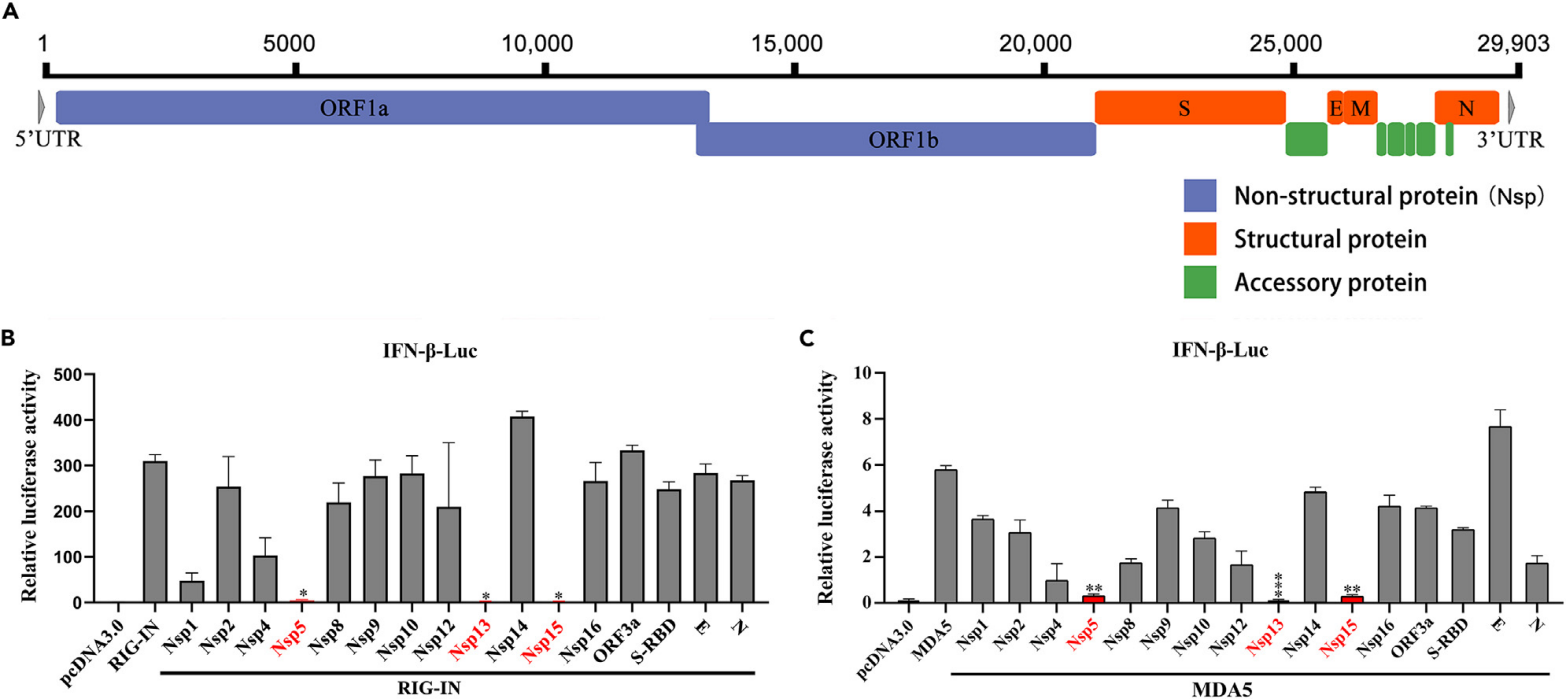
Screening of interferon antagonists among SARS-CoV-2 viral proteins (A) Genome architecture of SARS-CoV-2. (B and C) Screening of SARS-CoV-2 interferon antagonists. HEK-293T cells were co-transfected with pIFN-β-Luc together with the pRL-TK plasmids, expression plasmid for one of the indicated SARS-CoV-2 viral protein, and expression plasmid for RIG-IN (B) or MDA5 (C). At 24 h post-transfection, cells were lysed and dual luciferase activity was measured. Three independent experiments were done, and significance was calculated using Student’s two-tailed, unpaired *t* test. Error bars indicate SD of technical triplicates. ∗p < 0.05, ∗∗p < 0.01, and ∗∗∗p < 0.001.

**Figure 2:**
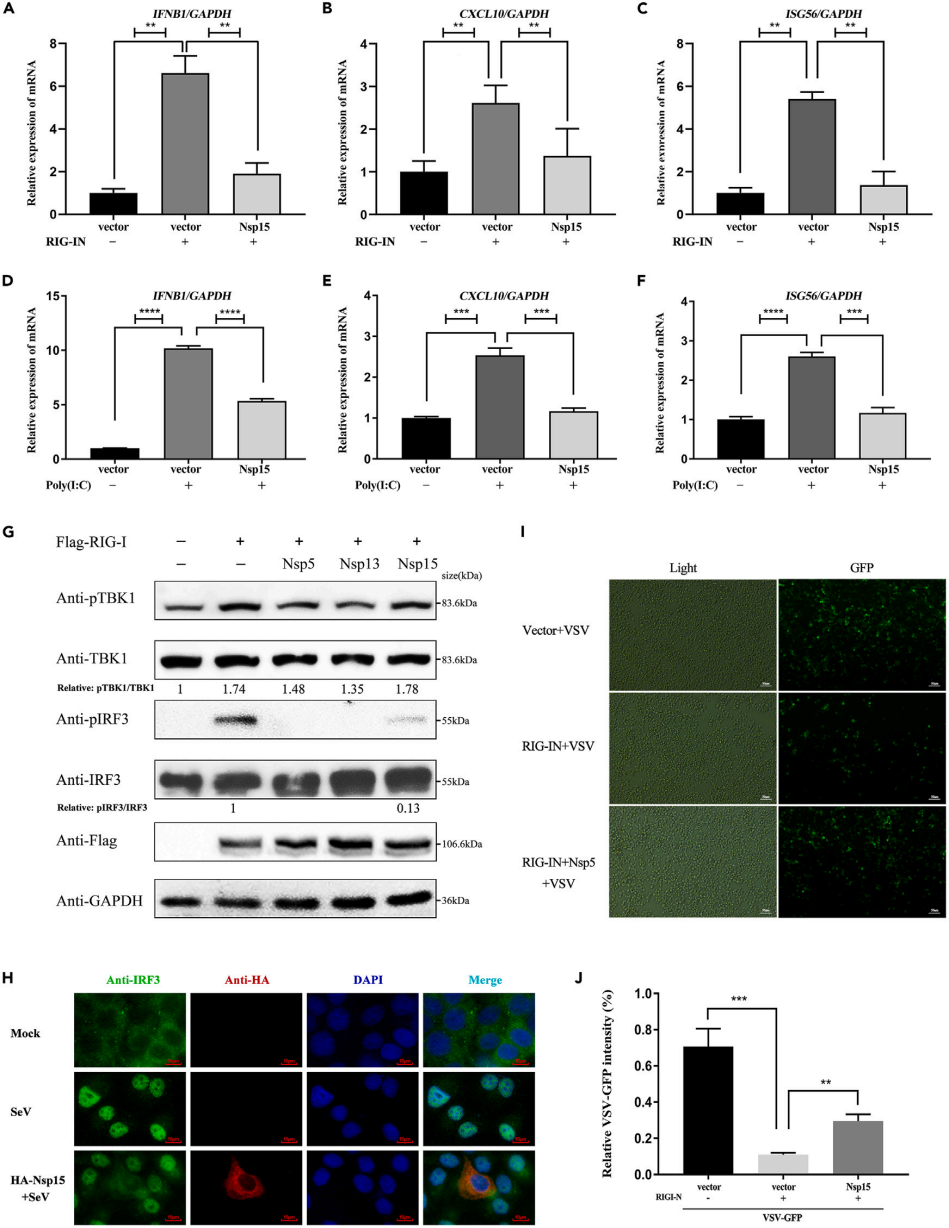
SARS-CoV-2 Nsp15 inhibits IRF3 phosphorylation and nuclear translocation (A–C) Relative mRNA levels of IFN-β and antiviral cytokine gene expression in stable Nsp15-expressing HEK-293T cells in response to RIG-IN induction were analyzed by qRT-PCR. GAPDH was used as a normalizer. (D–F) HEK-293T cells were transfected with empty plasmid or Nsp15 expressing plasmid. At 24 h post-transfection, cells were transfected with poly(I:C) for another 12 h. qRT-PCR was performed to determine IFN-I expression and antiviral cytokine expression. All data were expressed as mean ±SDs calculated from triplicate experiments (∗p < 0.05, ∗∗p < 0.01, ∗∗∗p < 0.001; two-tailed Student’s *t* test). (G) Effects of Nsp15 on homeostasis and phosphorylation of IRF3 and TBK1. HEK-293T cells were transfected with indicated Flag-tagged Nsp5, Nsp13, Nsp15 plasmid, together with RIG-I used as the stimulation plasmid. At 24 h post-transfection, the whole cell lysates (WCL) were detected with *p*-TBK1 (S172), TBK1, *p*-IRF3 (S396), and IRF3 antibodies, respectively. Relative levels of *p*-IRF3 and *p*-TBK1 were measured by ImageJ software. (H) HeLa cells were transfected with the control plasmid or the Nsp15 expression plasmid. After 24 h, the cells were infected with SeV for 12 h and then immunostained with the indicated antibodies. Images were obtained using an ortho fluorescence microscope. Green: endogenous IRF3 (488); Red: HA-Nsp15 (555); Blue: DAPI (nucleus). Scale bars, 10 μm. (I and J) RIG-IN and Nsp15-expressing plasmids were co-transfected into HEK-293T cells. After 24 h, the cells were subsequently infected with VSV-GFP for 10 h. Images were obtained with a fluorescent microscope. Scale bars, 50 μm. (I) and measured fluorescence intensity with ImageJ software (J). Three groups of data were measured repeatedly to calculate significance. All experiments were done at least twice, and one representative is shown. Data are presented with Means ± SD, ∗p < 0.05, ∗∗p < 0.01, ∗∗∗p < 0.001, two-tailed Student’s *t* test.

The SARS-CoV-2 genome encodes two large polyproteins, which consist of sixteen non-structural proteins (Nsps), four structural protein, and six accessory proteins (Figure 1A). To investigate the members of SARS-CoV-2 encoded proteins on IFN antagonism, we constructed 24 eukaryotic-expression plasmids encoding the SARS-CoV-2 genes. Western blot analysis revealed that 16 of these proteins were successfully expressed and detected, and their molecular weights matched the theoretical values (Figure S1A). However, eight viral proteins were not sufficiently expressed and therefore not included in the following study. We then used RIG-IN (the constitutively active N-terminal domains of RIG-I) and MDA5 as potent inducers for IFN production, and performed a dual-luciferase reporter assay to assess the level of interferon-beta (IFN-β) promoter induction. The results showed that three non-structural proteins (Nsp5, Nsp13, and Nsp15) significantly inhibited IFN-β-Luc activity (Figures 1B and 1C). Previous studies have revealed the mechanisms by which Nsp5 and Nsp13 regulate the type I IFN signaling pathway,^15^^,^^16^^,^^19^^,^^25^^,^^26^^,^^27^^,^^28^ hence, Nsp15 was chosen for further investigation. To gain a better understanding of the Nsp15-mediated inhibitory effect on the antiviral immune response, we further examined the expression levels of IFN-β and several ISGs. Using RIG-IN or poly(I:C) as agonists of the type I IFN signaling pathway, respectively, overexpressing Nsp15 significantly reduced the expression of *IFN-β*, IFN-stimulated gene 56 (*ISG56*), and cytokine *CXCL10* mRNA (Figures 2A–2F). These findings suggest that SARS-CoV-2 Nsp15 is a potential type I IFN antagonist.

### SARS-CoV-2 Nsp15 inhibits the phosphorylation and nuclear translocation of IRF3

TBK1 phosphorylation and IRF3 phosphorylation are two key steps in promoting the type I IFN signaling pathway. To investigate whether Nsp15 influences the phosphorylation and steady-state levels of TBK1 or IRF3, HEK-293T cells were co-transfected with plasmids expressing Nsp15 and RIG-IN. After 24 hpt, western blot analysis was conducted using the harvested cells. The results demonstrated that Nsp15 significantly suppressed 87% of IRF3 phosphorylation compared to when Nsp15 was absent, without significantly affecting TBK1 phosphorylation or overall TBK1 and IRF3 levels (Figure 2G, lane 5 compared to 2). Phosphorylated IRF3 can forms dimer, which then translocate into the nucleus to promote type I IFN production.^29^ These findings prompted us to investigate whether Nsp15 affects IRF3 nuclear translocation. Upon Sendai virus (SeV) infection, IRF3 was observed to noticeably translocate to the nucleus in the absence of Nsp15 expression, while SeV-mediated IRF3 nuclear translocation was reduced in cells expressing Nsp15 (Figure 2H).

Since Nsp15 potently suppresses IFN-β production, we further investigated whether Nsp15 has any role in regulating viral replication. GFP-VSV, an IFN-sensitive RNA virus strain, was chosen as the model virus to examine the effect of Nsp15 on viral infection and replication. Compared with the cells expressing the empty plasmid, the cells overexpressing RIG-IN could significantly reduce the fluorescence intensity of VSV-GFP, while the fluorescence intensity recovered obviously in the Nsp15-coexpressed cells (Figures 2I and 2J). These results indicated that RIG-IN could enhance the production of type I IFN protein, which inhibit the replication of GFP-VSV, but this effect could be suppressed by Nsp15, allowing for the proliferation of GFP-VSV in cells. Taken together, we have confirmed that Nsp15 is a key protein of SARS-CoV-2 in escaping host’s innate immune response via suppressing type I IFN production.

### SARS-CoV-2 Nsp15 interacts with TBK1 not IRF3

**Figure 3:**
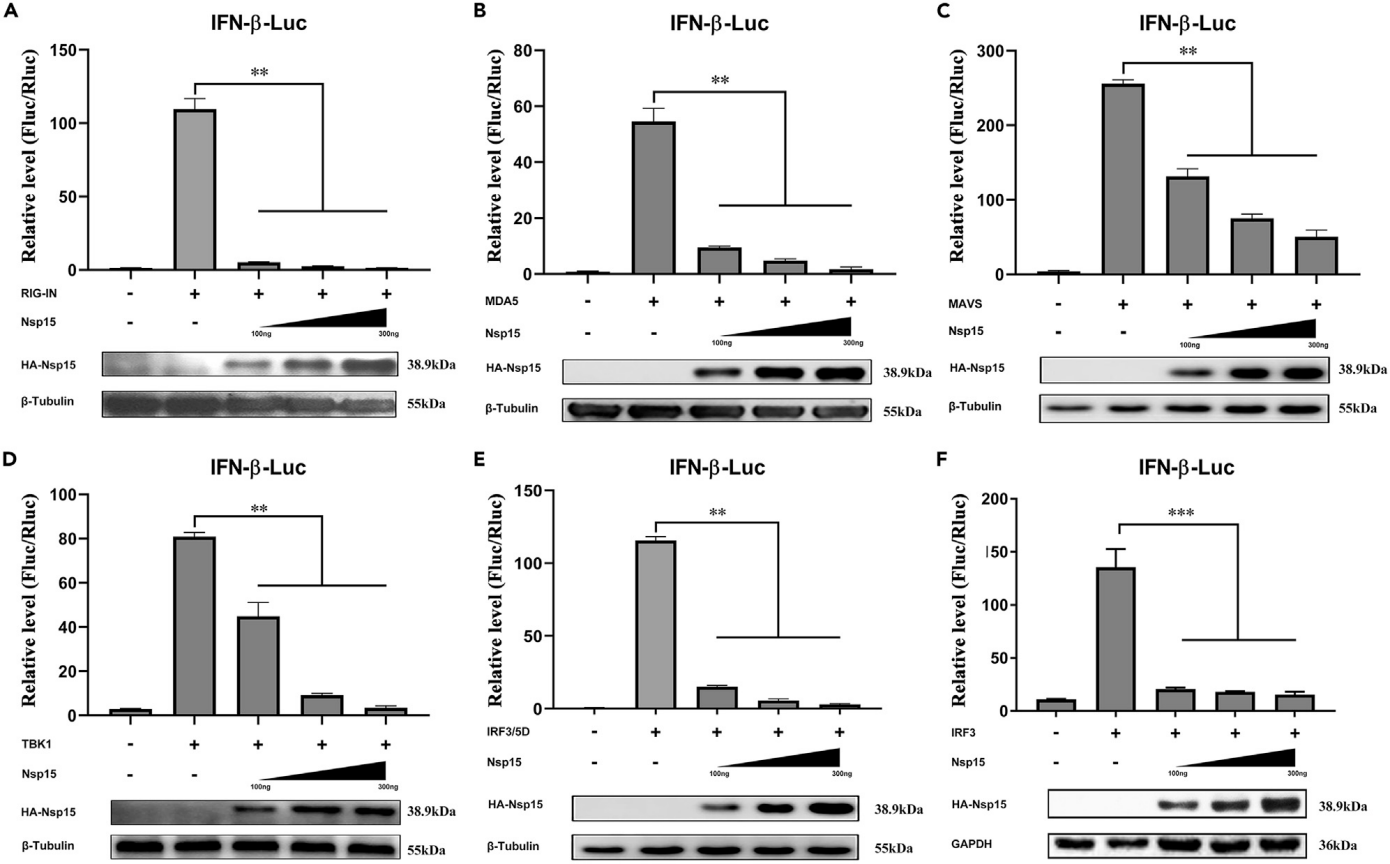
SARS-CoV-2 Nsp15 inhibits RLR-induced IFN-I activation (A–F) HEK-293T cells were co-transfected with pIFN-β-Luc, pRL-TK, and with increasing amounts of Nsp15-expressing plasmid, together with plasmids expressing RIG-IN (A), MDA5 (B), MAVS (C), TBK1 (D), IRF3/5D (E), and IRF3 (F). Equivalent amounts of lysates were also used for western blot analysis. All experiments were performed at least three times. Error bars indicate SDs from three independent experiments, ns represents non-significant, ∗p < 0.05, ∗∗p < 0.01, ∗∗∗p < 0.001, one-way ANOVA.

To clarify which steps in IFN-β production are regulated by Nsp15, we screened different key signaling molecules of the RLR pathway (RIG-IN, MDA5, MAVS, TBK1, IRF3 and its phosphor-mimic IRF3/5D) using dual-luciferase reporter assay. The results showed that overexpressed Nsp15 (at 100 ng, 200 ng, and 300 ng) significantly inhibited RIG-IN, MDA5, MAVS, TBK1, IRF3, and IRF3/5D-mediated activation of IFN-β promoter in a dose-dependent manner (Figures 3A–3F). Consistent with these findings, Nsp15 also decreased the activity of the IFN promoter induced by IRF7 (Figure S3). However, we did not include IRF7 in the current study due to its limited intracellular abundance and its primary function during the later stages of viral infection.^30^^,^^31^^,^^32^ Based on the results showing reduced nuclear translocation of IRF3 (Figure 2H), we speculated that Nsp15 antagonized IFN-β production by inhibiting the nuclear transport process of IRF3. Furthermore, since overexpressed Nsp15 significantly reduced the level of phosphorylated IRF3 (Figure 2G), it was suggested that Nsp15 could suppress IFN-β production by targeting IRF3 or another component upstream of IRF3.

**Figure 4:**
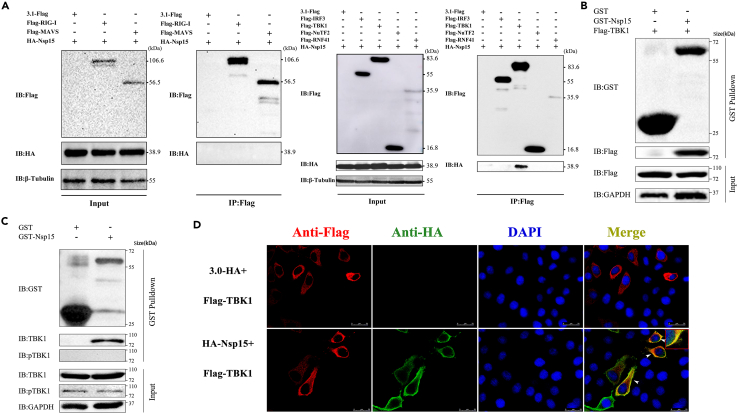
SARS-CoV-2 Nsp15 interacts with TBK1 (A) The plasmids of Flag-tagged RIG-I, MAVS, IRF3, TBK1, NuTF2, RNF41 and HA-Nsp15 were co-transfected into HEK-293T cells, and 24 h later, co-immunoprecipitation was performed using an anti-Flag Affinity Gel. (B and C) Purified GST-Nsp15 protein was incubated with Flag-TBK1 overexpressed or un-transfected HEK-293T cell lysates, and GST-Nsp15 interaction with Flag-TBK1 (B) or endogenous TBK1 (C) was analyzed by Western blot. (D) HeLa cells were transfected with the HA-Nsp15 and Flag-TBK1 expression plasmids for 24 h. Cells were immobilized and immunostained for detection of Nsp15 (Anti-HA, green) and TBK1 (Anti-Flag, red), and the nucleus was stained with DAPI (blue). Scale bar, 25 μm. All experiments were performed at least three times.

To further verify our conjecture, we performed the Co-IP assay by co-expressing Nsp15 with RIG-I, MAVS, TBK1, IRF3, RNF41, and NuTF2 in HEK-293T cells. RNF41 and NuTF2 were identified as Nsp15 interacting proteins by affinity-purification-mass spectrometry in a provirus study.^33^ The results revealed that Nsp15 only formed co-precipitation with TBK1, but not with others (Figure 4A). To further confirm the interaction of TBK1 and Nsp15, a GST-pull down assay was performed. The results showed that the prokaryotic-expressed GST-tagged Nsp15 coprecipitated both the overexpressed and endogenous TBK1 in HEK-293T cells, but not the GST protein itself (Figures 4B and 4C). Furthermore, the immunofluorescence assay demonstrated that Nsp15 (Green) and TBK1 (Red) were clearly observed homologation signals in the cytoplasm (Figure 4D). Collectively, these findings show that Nsp15 interacts with TBK1, but not with IRF3.

### The KD domain of TBK1 is required for interaction with Nsp15

**Figure 5:**
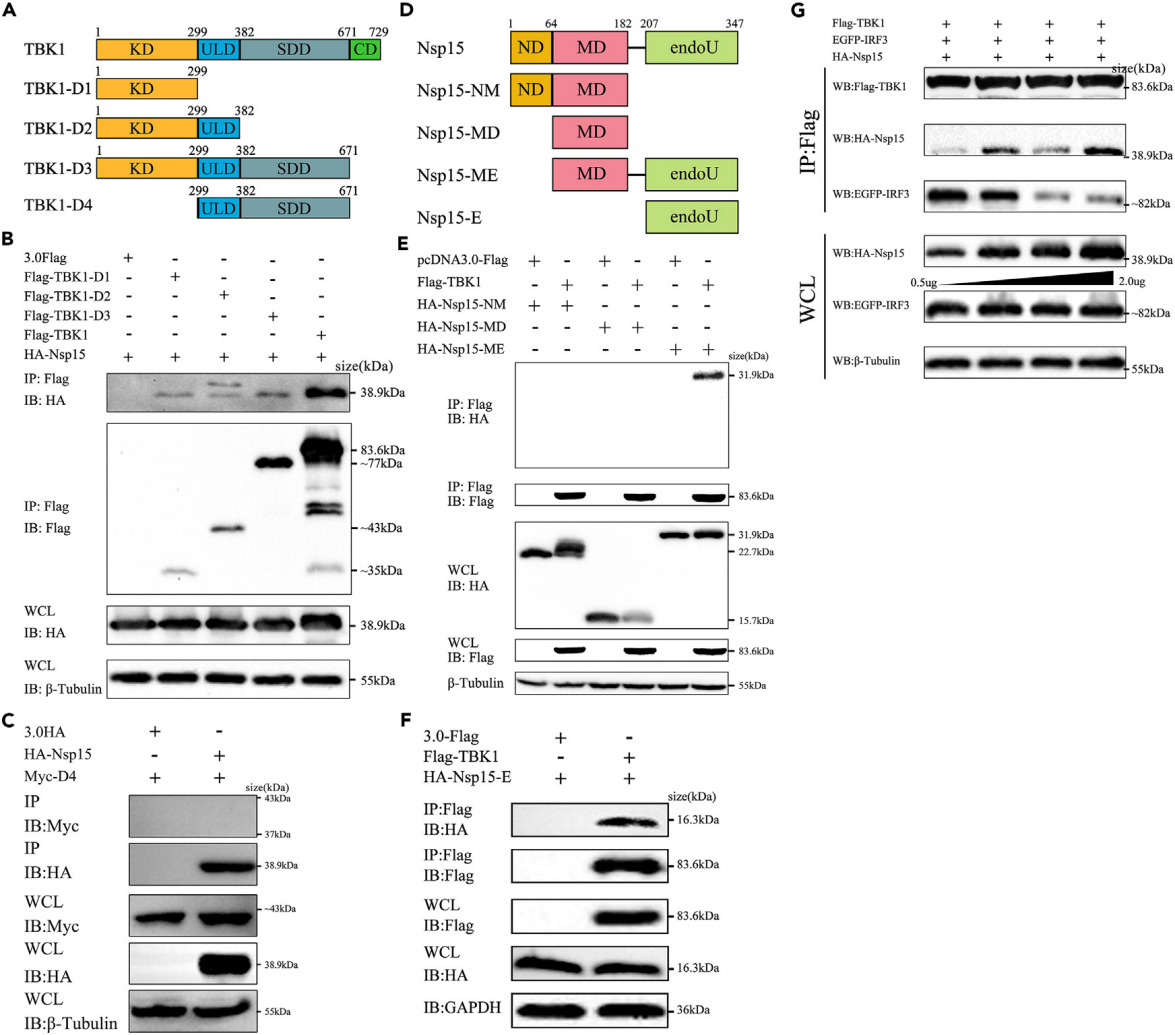
The EndoU domain of SARS-CoV-2 Nsp15 is required for interaction with TBK1 (A) Schematic representation of TBK1 domain positions. KD: kinase domain; ULD: ubiquitin-like domain; SDD: scaffold dimerization domain; CD: C-terminal domain. (B and C) HEK-293T cells were transfected with Flag-tagged TBK1 truncated fragments and HA-Nsp15, and co-immunoprecipitation and immunoblot analysis were performed after 24 h. (D) Schematic representation of SARS-CoV-2 Nsp15 domains according its amino acid sequence. ND: N-terminal domain; MD: middle domain; EndoU: poly-U-specific endonuclease domain. (E and F) HEK-293T cells were transfected with HA-tagged Nsp15 truncated fragments and Flag-TBK1 for 24 h. The WCL and immunoprecipitates were immunoblotted with the indicated antibodies. (G) Nsp15 impedes the formation of TBK1/IRF3 complex. HEK-293T cells were co-transfected with Flag-TBK1, EGFP-IRF3, together with increasing doses of HA-Nsp15 for 24 h. Cell lysates were immunoprecipitated with anti-Flag Affinity Gel. The WCL and immunoprecipitates were immunoblotted with the indicated antibodies. All experiments were performed at least twice.

To explore the major domain of TBK1 that interacts with Nsp15, three truncated TBK1 clones were constructed named TBK1-D1, TBK1-D2, and TBK1-D3. TBK1-D1 contained only the kinase domain (KD), TBK1-D2 consisted of KD and the ubiquitin-like domain (ULD), and TBK1-D3 included KD, ULD, and the scaffold dimerization domain (SDD)^34^ (Figure 5A). The molecular weight of each truncated protein corresponded with the prediction (Figure S1B). We co-expressed HA-Nsp15 with Flag-TBK1 or the truncated forms in HEK-293T cells. We then performed co-IP using an Anti-Flag antibody. As depicted in Figure 5B, TBK1 and all three truncations displayed interaction with Nsp15. Additionally, we constructed a truncated form of TBK1 that lacked the KD. Co-immunoprecipitation experiments failed to detect any binding between Nsp15 and this truncated D4 form (Figure 5C). It suggests that the KD of TBK1 is required for its interaction with Nsp15.

### The EndoU domain of Nsp15 mediates its interaction with TBK1

Nsp15 is a uridine-specific endoribonuclease containing three major domains: the N-terminal domain (ND), the middle domain (MD), and the poly-U-specific endonuclease domain (EndoU). To determine which domain of NSP15 binding to TBK1, we constructed four Nsp15 truncated mutations into the eukaryotic expression vector. These truncations included NM (ND + MD, 1-206aa, 22.7 kDa), MD (64-206aa, 15.7 kDa), ME (MD + EndoU, 64-347aa, 31.9 kDa), and E (EndoU, 207-347aa, 16.3kDa) (Figure 5D). The expression of each truncation was confirmed using western blot analysis (Figure S1C). HEK-293T cells were co-transfected Flag-TBK1 with HA-Nsp15 or the four truncations, respectively. Co-IP was then performed to target Flag-TBK1, followed by western blotting to detect the presence of full length or truncated Nsp15 in the immunoprecipitates. The results showed that TBK1 precipitated with the full length, ME, and E truncations of Nsp15, but not NM and MD truncations (Figures 5E and 5F). This indicates that the EndoU domain of Nsp15 plays a critical role in its interaction with TBK1.

### SARS-CoV-2 Nsp15 interferes the interaction between TBK1 and IRF3 by competitive combining with TBK1

TBK1, as a protein kinase, plays an essential role in the phosphorylation and activation of IRF3.^10^ Since Nsp15 interacted with TBK1 but not IRF3 and had no apparent effect on TBK1 phosphorylation or total IRF3 and TBK1 levels (Figures 2G and 4A), we further investigated whether Nsp15 affected the interaction between TBK1 and IRF3. HEK-293T cells were transfected with Flag-TBK1, GFP-IRF3, and increasing dose of HA-Nsp15 plasmids (0.5 μg, 1.0 μg, 1.5 μg, 2.0 μg) for immunoprecipitation. Co-IP results showed that Nsp15 weakened the interaction between TBK1 and IRF3 in a dose-dependent manner (Figure 5G). It indicates that Nsp15 competitively binds to TBK1, thereby blocking IRF3 phosphorylation and antagonizing type I IFN production.

### SARS-CoV-2 Nsp15 inhibits IRF3 nuclear translocation via binding and degrading KPNA1

**Figure 6:**
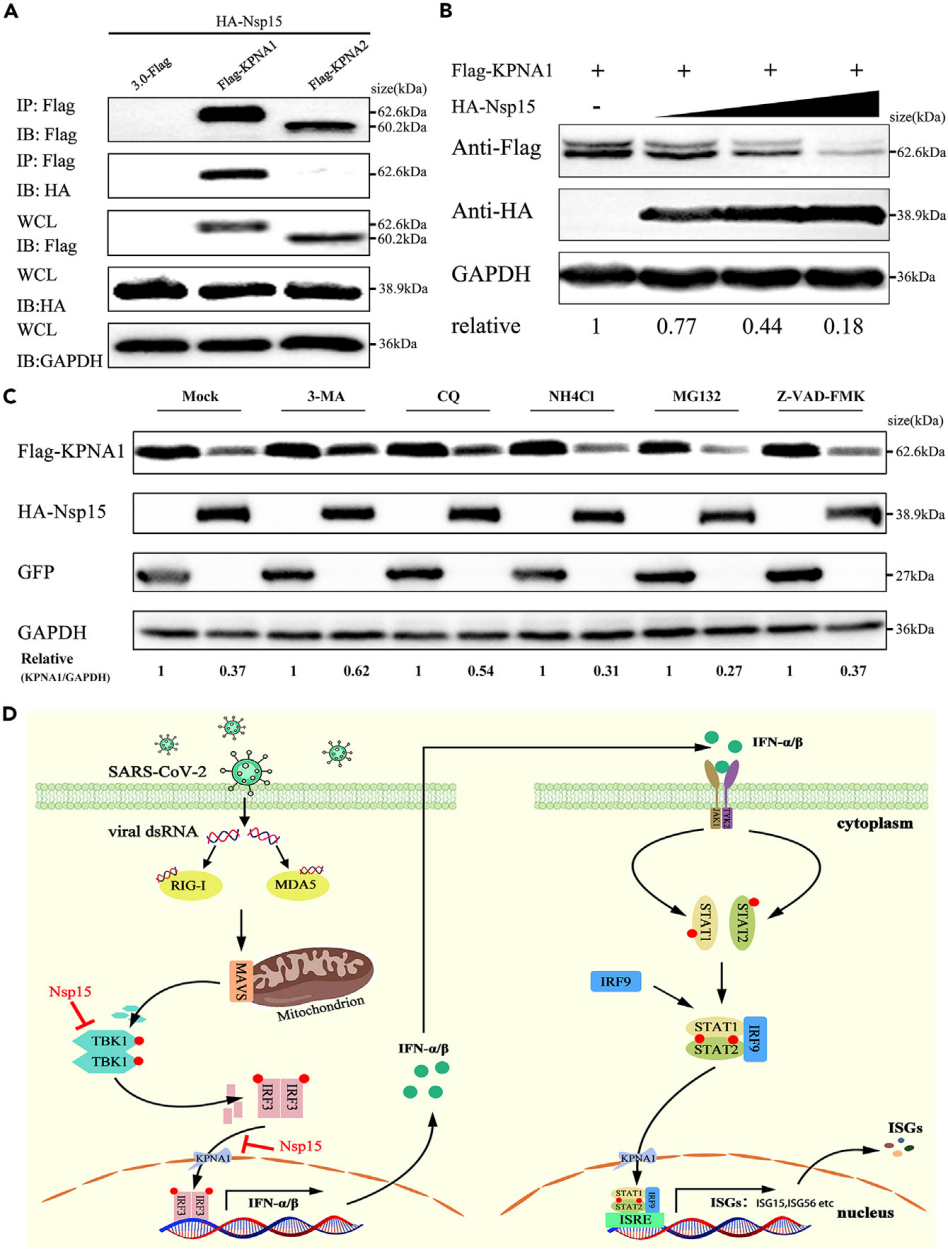
SARS-CoV-2 Nsp15 bindings and reduces KPNA1 protein in a dose-dependent manner (A) HEK-293T cells were transfected with HA-Nsp15, along with Flag-KPNA1 or Flag-KPNA2 for 24 h. Cell lysates were immunoprecipitated with anti-Flag Affinity Gel and then immunoblotted with the indicated antibodies. (B) HEK-293T cells were transfected with increasing doses of HA-Nsp15 expressing plasmid, along with Flag-KPNA1 plasmid for 24 h. The WCL were immunoblotted with the indicated antibodies. (C) HEK-293T cells were transfected with plasmids containing KPNA1 and Nsp15. After 24 h, the cells were treated with lysosome inhibitor (3-MA, CQ, and NH_4_Cl), proteasome inhibitor (MG132), or pan-caspase inhibitor (Z-VAD-FMK), followed by western blot analysis. (D) Schematic depiction of Nsp15 inhibition of the RLR signaling pathway. All experiments were performed at least three times.

Karyopherins are a group of proteins that mediate the nucleocytoplasmic trafficking of numerous proteins, including transcription factors of the innate immune system.^35^^,^^36^^,^^37^ There are seven isoforms of karyopherins, namely karyopherin α1 to α7 (KPNA1∼KPNA7).^38^ It has been proven that KPNA1 and KPNA2 participate in the IRF3 nuclear transfer process.^39^^,^^40^ Hence, we further explored whether Nsp15 inhibits IRF3 nuclear translocation by hijacking KPNA1 or KPNA2. To verify our conjecture, we co-transfected HA-Nsp15 and Flag-KPNA1 or Flag-KPNA2 into HEK-293T cells. The results of co-IP showed that Nsp15 could be co-precipitated with KPNA1, but not with KPNA2 (Figure 6A). Further study discovered that overexpressed Nsp15 (150 ng, 300 ng, and 600 ng) reduced KPNA1 protein levels in a dose-dependent manner (Figure 6B). To explore the mechanism of Nsp15-mediated KPNA1 degradation, we co-expressed KPNA1 and Nsp15 plasmids into cells and treated them with autophagy pathway inhibitors (3-MA, CQ and NH_4_Cl), proteasome inhibitors (MG132), and pan-caspase inhibitor (Z-VAD-FMK), respectively. The results showed that 3-MA and CQ restored KPNA1 expression levels to some extent, while NH_4_Cl, MG132, and Z-VAD-FMK did not inhibit KPNA1 degradation mediated by Nsp15 (Figure 6C). These findings confirmed that Nsp15 binds to KPNA1 and induces its degradation via the autophagy-lysosomal pathway, further inhibiting the translocation of phosphorylated IRF3 into the nuclear, which antagonizes type I IFN production.

## Discussion

During a viral infection, type I IFN responses serve as the first line of antiviral immunity to inhibit viral proliferation. Clinical symptoms in COVID-19 patients and studies in animal models or human cells have shown that SARS-CoV-2 only weakly induces type I IFN response, suggesting that it may have developed a more efficient way of surviving in its host. Previous reports have revealed that SARS-CoV-2 Nsp15 protein has the ability to counteract host’s innate immune response.^17^^,^^19^^,^^24^ However, the specific molecular mechanism of Nsp15 on suppressing host type I IFN production remains unclear. In the present study, we confirmed Nsp15 as an antagonist of RLR with two potential regulatory mechanisms to suppress type I IFN production (Figure 6D).

Nsp15, a nidoviral RNA uridylate-specific endoribonuclease (NendoU), is conserved in coronaviruses.^41^ Previous research has shown that MHV Nsp15 can antagonize antiviral innate immune responses by cleaving viral dsRNA, thus avoiding recognition by dsRNA sensors.^42^ Porcine deltacoronavirus Nsp15 antagonizes IFN responses independently of its endoribonuclease activity.^43^ These findings suggest that different coronavirus Nsp15 may have different mechanism in antagonizing IFN responses. Our present study newly found that SARS-CoV-2 Nsp15 can also antagonize host innate immunity by directly interacting with TBK1 (Figure 4), which is a key signal protein in RLR-inducing IFN production axis.^41^^,^^43^ The KD domain of TBK1 was identified as the crucial region for their interaction. Studies have explored that autophosphorylation at Ser-172 in the KD domain can activate TBK1 kinase, and this step is essential for virus-triggered signaling.^44^ Surprisingly, Nsp15 binds to the TBK1 KD domain neither masking the Ser-172 phosphorylation, nor blocking TBK1 activity (Figures 2G and 4). This indicates that the interaction between Nsp15 and TBK1 might suppress the downstream signaling transduction of TBK1, but not the activation of TBK1. IRF3 is a direct phosphorylated substrate of TBK1, which depend on their recruited via MAVS/STING/TRIF protein proximity to each other.^45^ Our result testified that Nsp15 weakens the interaction between TBK1 and IRF3 by competitively binding to TBK1, thereby reducing the phosphorylation of IRF3 (Figure 5G). Therefore, we conclude that although SARS-CoV-2 Nsp15 does not change the phosphorylation activation of TBK1, it may change the spatial distance between TBK1 and IRF3, causing TBK1 to be unable to activate IRF3.

Moreover, the EndoU domain of SARS-CoV-2 Nsp15, which includes the catalytic triad (His235, His250, and Lus290) is essential for its endonuclease activity.^41^ Loss of Nsp15 enzyme activity in porcine epidemic diarrhea virus (PEDV), mouse hepatitis virus (MHV), and human coronavirus 229E (HCoV-229E) activated IFN responses and reduced viral titers.^21^^,^^46^ Previous study has shown that PEDV Nsp15 can directly degrade the RNA levels of TBK1 and IRF3 dependent on its EndoU activity.^47^ In the present study, we have discovered that SARS-CoV-2 Nsp15 interacts with TBK1 via its EndoU domain (E), but not ND and MD domains (Figures 5E and 5F). Additionally, ME and E truncations alone inhibited TBK1-induced IFN expression by 44.8% and 67.2%, respectively (Figure S2). These findings indicate that the EndoU domain of Nsp15 plays a vital role in regulating the production of IFN-β. Conducting further research on whether the endonuclease activity affects IFN expression will greatly enhance our understanding of the mechanism by which Nsp15 regulates the RLR signaling pathway. It is worth noting that the hexamerization of Nsp15 is critical for its endoribonuclease activity, while the ND domain of Nsp15 is essential for the formation of hexamer.^48^^,^^49^ Given that the ND domain of SARS-CoV-2 Nsp15 also inhibits TBK1-mediated IFN-β promoter activation in our study, we hypothesize that the hexamer formed by the dominant ND domain can inhibits the binding of TBK1 and IRF3 through spatial occupation.

The nuclear translocation of phosphorylated IRF3 is necessary for inducing type I IFN production. Karyopherins (KPNAs) are essential transporters that recognize specific nuclear localization signals or nuclear export signals to facilitate the transport of proteins in and out of the nucleus.^50^^,^^51^ Many viruses also target KPNAs to evade host antiviral responses. For example, the Zika virus NS2A protein induced KPNA2 degradation through chaperone-mediated autophagy to ensure efficient viral replication in infected cells.^52^ PEDV Nsp7 inhibited JAK-STAT signaling by blocking ISGF3 nuclear translocation by binding to KPNA1.^36^ Despite SARS-CoV-2 Nsp15 interfering with the interaction between TBK1 and IRF3, it still does not explain why Nsp15 can block the activation IRF3-inducing IFN production independent on binding to IRF3 (Figures 3E and 4A). Here, we identified that SARS-CoV-2 Nsp15 as a newly discovered KPNA1 interacting viral protein. SARS-CoV-2 Nsp15 could reduce its expression in a dose-dependent manner (Figure 6B). Treatment with autophagy inhibitors 3-MA and CQ, significantly restored KPNA1 protein expression, while NH4Cl did not have the same effect. Furthermore, treatment with proteasome inhibitor MG132 and pan-caspase inhibitor Z-VAD-FMK did not restore KPNA1 protein expression levels (Figure 6C). 3-MA inhibited autophagosome formation, CQ inhibited autophagosome-lysosome fusion, and NH4Cl inhibited autophagosome degradation through increasing intracellular PH of lysosomes. Therefore, we speculated that Nsp15 might function in the early stages of autophagy. This is consistent with previous reports that Nsp15 can block autophagosome formation.^53^ Jiang et al. reported that upregulation of KPNA1 effectively promoted IRF3 and ISGF3 nuclear translocation.^39^ Cai et al. testified that KPNA2 promoted virus-induced IRF3 nuclear translocation and cellular antiviral responses.^40^ The evidence mentioned above indicates that phosphorylated IRF3 is dependent on associating with KPNA1/2 to be imported into the nuclear. Since IRF3 acted as a KPNA1 trafficking substrate, we suspected that SARS-CoV-2 Nsp15 is hijacking and promoting autophagy-dependent degradation of KPNA1 to block the nuclear translocation of phosphorylated IRF3. This represents another strategy of SARS-CoV-2 Nsp15 to further suppress the RLR-mediated type I IFN production.

In summary, we have identified SARS-CoV-2 Nsp15 as a significant inhibitor of host RLR-mediated type I IFN production through its interaction with TBK1 and KPNA1, inhibiting IRF3 phosphorylation and nuclear translocation, respectively (Figure 6D). Admittedly, there are still some limitations in our study. In the future, more attention should be paid to the function of Nsp15 under the condition of SARS-CoV-2 infection. It is worth determining whether SARS-CoV-2 Nsp15 promotes KPNA1 autophagy degradation is dependent on the polyubiquitination of KPNA1 and which autophagy receptor is involved in this process. Clarifying the answers to these inquiries will improve our understanding of the molecular mechanism by which SARS-CoV-2 Nsp15 inhibits IFN expression. Even so, our study provided new insights into the evasion of host innate immunity by SARS-CoV-2.

### Limitations of the study

First, host antiviral immunity is a multidirectional and complex regulatory process. Simulating the true immune escape mechanism of a virus in a single cell line is difficult, and experimental results may not necessarily reflect real changes *in vivo*. Second, studies on Nsp15 mainly rely on plasmids to activate a single IFN pathway, which does not accurately reflect the functional performance of Nsp15 during SARS-CoV-2 infection. Furthermore, the mechanism of Nsp15 can be further confirmed by mutant Nsp15-deficient virus strains to infect cells or by constructing infected animal models. Furthermore, the precise mechanism underlying Nsp15’s regulation of KPNA1 involvement in autophagy requires further exploration. This investigation will contribute to a more comprehensive understanding of the pathogenesis of SARS-CoV-2.

## STAR★Methods

### Key resources table

**Table:** 

REAGENT or RESOURCE	SOURCE	IDENTIFIER
**Antibodies**		

IRF-3 (D6I4C) XP® Rabbit mAb	Cell Signaling Technology	Cat#11904S; RRID: AB_2722521
Phospho-IRF-3 (Ser396) (4D4G) Rabbit mAb	Cell Signaling Technology	Cat#4947S; RRID: AB_823547
TBK1/NAK (E8I3G) Rabbit mAb	Cell Signaling Technology	Cat#38066S; RRID: AB_2827657
Phospho-TBK1/NAK (Ser172) (D52C2) XP® Rabbit mAb	Cell Signaling Technology	Cat#5483S; RRID: AB_10693472
DYKDDDDK Tag (D6W5B) Rabbit mAb	Cell Signaling Technology	Cat#14793S; RRID: AB_2572291
HA tag Rabbit Monoclonal Antibody	Cell Signaling Technology	Cat#5017; RRID: AB_10693385
GST Mouse Monoclonal Antibody	Sigma-Aldrich	Cat#G1160; RRID: AB_259845
Myc Tag Mouse Monoclonal Antibody	Beyotime	Cat#AF0033; RRID: AB_2939055
GFP Rabbit Monoclonal Antibody	Cell Signaling Technology	Cat#2956; RRID: AB_1196615
Alexa Fluor 488-labeled Goat Anti-Rabbit IgG(H + L)	Beyotime	Cat#A0423; RRID: AB_2891323
Alexa Fluor 555-labeled Donkey Anti-Mouse IgG(H + L)	Beyotime	Cat#A0460; RRID: AB_2890133
GAPDH Rabbit mAb	ABclonal	Cat#A19056; RRID: AB_2862549
β-Tubulin Rabbit mAb	ABclonal	Cat#A12289; RRID: AB_2861647
HRP Rabbit Anti-Goat IgG (H + L)	ABclonal	Cat#AS029; RRID: AB_2769859
HRP Goat Anti-Mouse IgG (H + L)	ABclonal	Cat#AS003; RRID: AB_2769851

**Bacterial and virus strains**		

Sendai virus	This paper	N/A
recombinant VSV-GFP strains	This paper	N/A

**Chemicals, peptides, and recombinant proteins**		

Lipo6000™ Transfection Reagent	Beyotime	Cat#C0526;
PMSF	Beyotime	Cat#ST507;
RIPA Lysis Buffer	Beyotime	Cat#P0013D
5×SDS-PAGE Protein Loading Buffer	YEASEN	Cat#20315ES05
GoldBand Plus 3-color Regular Range Protein Marker(8–180 kDa)	YEASEN	Cat#20350ES72
GSTSep Glutathione Agarose Resin	YEASEN	Cat#20507ES10;
InStab™ Phosphatase Inhibitor Cocktail	YEASEN	Cat#20109ES05;
Anti-Flag Affinity Gel	Bimake	Cat#B23101;
Protease Inhibitor Cocktail	Merck	Cat#P8340;

**Critical commercial assays**		

RevertAid First Strand cDNA Synthesis Kit	ThermoFisher Scientific	Cat#K1622;
Dual Luciferase Reporter Assay Kit	Vazyme	Cat#DL101-01;
RNA-easy Isolation Reagent	Vazyme	Cat#R701-01;
ChamQ Universal SYBR qPCR Master Mix	Vazyme	Cat#Q711-02;

**Experimental models: Cell lines**		

HEK-293T	ATCC	N/A
HeLa	ATCC	N/A

**Oligonucleotides**		

Primers for sequences, see Table S1	This paper	N/A

**Recombinant DNA**		

pcDNA3.0-HA	This paper	N/A
pcDNA3.0-Flag	This paper	N/A
pEGFP-C1	This paper	N/A
pET-28a+	This paper	N/A
pGL3-IFN-β-Luc	This paper	N/A
pRL-TK	This paper	N/A

**Software and algorithms**		

ImageJ	National Institutes of Health	Version 1.8.0
GraphPad Prism	GraphPad Software	Version 7.04
LAS AF Lite	Leica microsystems	Version 2.3.5
Geneious Prime	Biomatters	Version 2020.05

### Resource availability

#### Lead contact

Further information and requests for reagents may be directed to and will be fulfilled by the corresponding author Prof. Shixing Yang (johnsonyang1979@163.com).

#### Materials availability

This study did not generate new unique reagents. Requests for the plasmids, cell lines, and antibodies generated in this study should be directed to the lead contact with a completed Materials Transfer Agreement.

### Experimental model and subject details

#### Cell lines and virus stains

HEK-293T and HeLa cells were purchased from the American Type Culture Collection (ATCC). These cells were cultured in Dulbecco’s modified Eagle medium (DMEM, HyClone) supplemented with 10% heat-inactivated fetal bovine serum (FBS, Gibco), 2 mM glutamine, 100 U/ml penicillin, and 100 U/ml streptomycins at 37°C in 5% CO2 humidified atmosphere. The cells in the logarithmic growth state were tested for mycoplasma (Vazyme, Cat#D101-01), and the cells without contamination were frozen or further cultured. The cells were transfected with related plasmids by lipo6000 (Beyotime, Cat#C0526). 24 h later, plasmids were stably expressed in cells by Western blot and used for further experiments. The Sendai virus (SeV) and recombinant VSV-GFP strains were originally preserved in our laboratory and virus titers were determined by TCID50.

#### Plasmids

The 24 prokaryotic expression plasmids of SARS-CoV-2 genes were kindly provided by Shengce Tao (Shanghai Jiaotong University) and further cloned into the pcDNA3.0+ vector with an HA-tag at the N terminus (pcDNA3.0-HA) by us. Nsp15 and its truncations were constructed in the pcDNA3.0-HA vector. RIG-IN (the constitutively active N-terminal domains of RIG-I), MDA5, MAVS, TBK1, IRF3, IRF3/5D, and TBK1 truncations were cloned into pcDNA3.0+ vector with the FLAG tag at the N-terminus. The human IRF3 was cloned into pEGFP-C1 vector. The pGL3-IFN-β-Luc and pRL-TK plasmids were preserved in our laboratory. Nsp15 was cloned into the reconstructed pET-28a+ vector with glutathione S-transferase (GST) protein.

### Method details

#### Dual-luciferase reporter assay

The luciferase reporter (pGL3-IFN-β-Luc) and the Renilla luciferase reporter (pRL-TK) plasmids, along with the indicated expression plasmids, were transfected into HEK-293T cells by Lipo6000 Transfection Reagent (Beyotime, China). After 24 h transfection, cell lysates were used for detecting luciferase activity using a Dual Luciferase Reporter Assay Kit (Vazyme, Cat#DL101-01), and the firefly luciferase activities were normalized to Renilla luciferase activities.

#### RNA extraction, reverse transcription, and quantitative real-time PCR

Total RNA was extracted from cultured cells with TRIzol reagent (Vazyme, Cat# R701-01), reverse transcribed into cDNA with reverse transcriptase (Thermo, Cat# K1622). cDNAs were prepared for the real-time PCR by using SYBR Green PCR mix (Vazyme, Cat#Q711) with ABI 7500 system (USA).

#### Immunofluorescence

HeLa cells seeded on chamber-slides were fixed with 4% paraformaldehyde (Beyotime, Cat#P0099). Then cells were permeablilized with 0.5% Triton X-100 (Beyotime, Cat#P0096). After cells were washed for three times with PBS, they were blocked for 1 h with TBST containing 5% BSA. Cells were then incubated separately for 4 h at 37°C with either rabbit monoclonal antibody against FLAG tag (1:500) or mouse monoclonal antibody against HA-tag (1:1000), followed by staining with an Alexa Fluor 488 (Beyotime, Cat#A0423) or an Alexa Fluor 555 (Beyotime, Cat#A0460) secondary antibodies. Nuclei were stained for 1 min at room temperature with DAPI (Beyotime, Cat#C1006). A confocal laser scanning microscope was used to visualize and obtain fluorescent images, and images were prepared using the LAS AF Lite software (Version 2.3.5).

#### Co-immunoprecipitation (Co-IP) and immunoblotting

For co-IP assay, HEK-293T cells were harvested with precooled RIPA lysis buffer (Beyotime, China) containing protease inhibitor PMSF (Beyotime, China) and phosphatase inhibitor cocktail (YEASEN, China). After centrifugation at 14,000×g for 10 min at 4°C, the supernatant was immunoprecipitated against anti-Flag Affinity Gel for 4 h at 4°C. Then, the beads were washed with 1×TBS for three times before boiling with 1 × SDS-PAGE loading buffer (YEASEN, China) to elute the immunoprecipitate. Precipitated proteins were analyzed by SDS-PAGE gel electrophoresis and immunoblotting.

#### Virus replication assay

HEK-293T cells were transfected with an empty vector, RIG-IN-expressing plasmid or RIG-IN-expressing plasmid together with Nsp15-expressing plasmid, respectively. After 24 h post-transfection, cells were infected with VSV-GFP strain at the desired multiplicity of infection (MOI = 0.01) for 10 h. Virus replication of different groups were then observed using fluorescence microscopy. ImageJ was used to calculate fluorescence intensity.

#### GST precipitation

The Nsp15 gene was cloned into the pET-28(a)-GST vector and transformed into Escherichia coli BL21(DE3) (Vazyme, China) to express GST-tagged Nsp15. Protein interactions were examined with a GST protein interaction pulldown kit (YEASEN, Cat#20507ES10) by following the manufacturer’s instruction. Western blot assay was performed for protein analysis after elution using reduced glutathione.

### Quantification and statistical analysis

Statistical analyses were performed in GraphPad Prism 7. The two-tailed Student’s t-test was used for two-group comparisons. Two-way analysis of variance (ANOVA) was applied when more than two groups were compared. The values of p < 0.05 were considered statistically significant and p < 0.01 were considered statistically highly significant.

## Data Availability

• All data reported in this paper will be shared by the lead contact upon request. • This paper does not report original code. • Any additional information required to reanalyze the data reported in this work paper is available from the lead contact upon request.
