# Supplementary material for: SARS-CoV-2 Nsp15 suppresses type I interferon production by inhibiting IRF3 phosphorylation and nuclear translocation
Source: iScience. 2023 Aug 23;26(9):107705. doi: 10.1016/j.isci.2023.107705 (PMC10480782; doi:10.1016/j.isci.2023.107705)
Supplement: Document S1. Figures S1–S4 and Table S1 [file mmc1.pdf]

iScience, Volume *26*

Supplemental information

SARS-CoV-2 Nsp15 suppresses type I interferon production by inhibiting IRF3 phosphorylation and nuclear translocation

Dianqi Zhang, Likai Ji, Xu Chen, Yumin He, Yijie Sun, Li Ji, Tiancheng Zhang, Quan Shen, Xiaochun Wang, Yan Wang, Shixing Yang, Wen Zhang, and Chenglin Zhou

This file includes Figure S1 to S4, and Table S1

Supplementary Figure Legends

Figure S1

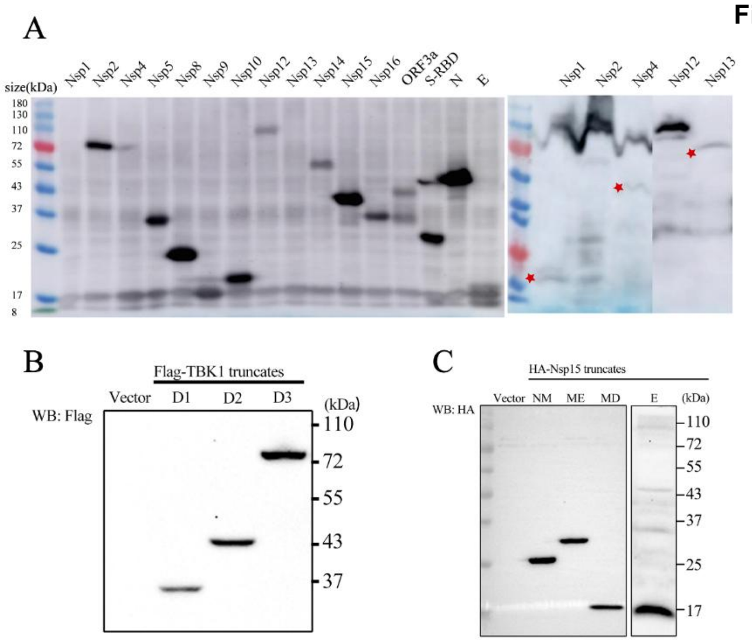

Figure S1. Expression of related proteins. Related to Figure 1 and Figure 5.

(A) HEK-293T cells were transfected with the indicated plasmid. After 24 h post-transfection, cells were harvested and immunoblotted with HA-tagged antibodies. (B) Expression of TBK1 truncated proteins. N-terminally Flag-tagged truncated proteins were expressed in HEK-293T cells for 24 h and analyzed by Western blot using the anti-Flag antibody. (C) Expression of Nsp15 truncated proteins. N-terminally HA-tagged truncated proteins were expressed in HEK-293T cells for 24 h and immunoblotted with the anti-HA antibody.

Figure S2

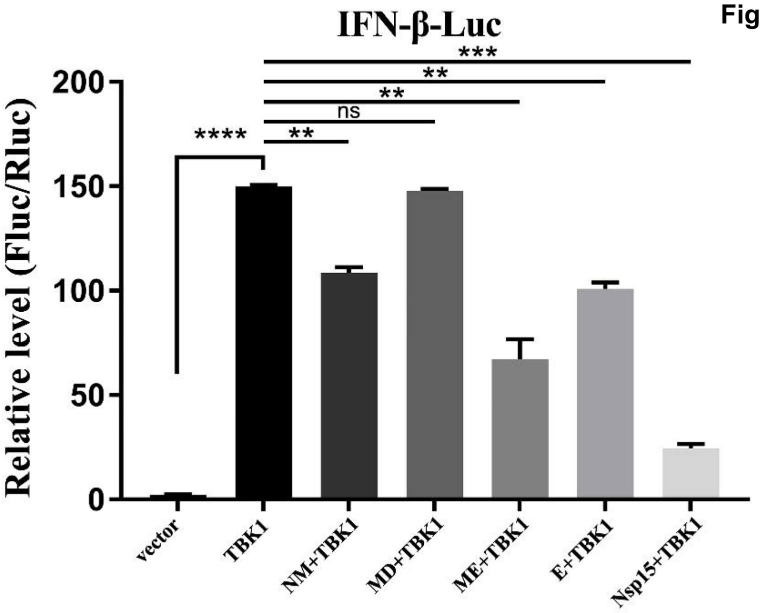

Figure S2. Screening of SARS-CoV-2 Nsp15 truncated proteins interferon antagonists. Related to Figure 5.

IFN-β-luc reporter plasmids were co-transfected with pRL-TK plasmids, TBK1 expression

vector and SARS-CoV-2 Nsp15 or its truncated protein plasmids into HEK-293T cells for 24 h, respectively. Cells were then lysed for dual-luciferase assay. The experiment was done at least three times, and error bars indicate SDs from three independent experiments, ns represents non-significant, * $p < 0.05$, ** $p < 0.01$, *** $p < 0.001$, two-tailed Student's t-test.

Figure S3

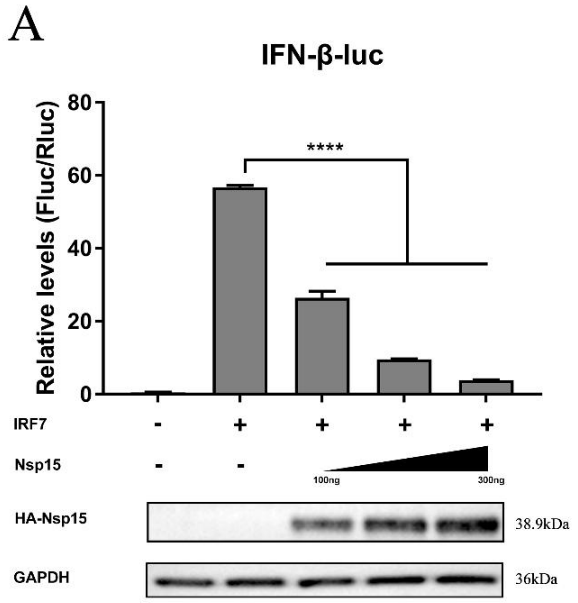

Figure S3. SARS-CoV-2 Nsp15 inhibits IRF7-induced IFN-I activation. Related to Figure 3.

(A) HEK-293T cells were co-transfected with pIFN-β-Luc, pRL-TK, and with increasing amounts of Nsp15-expressing plasmid, together with plasmids expressing IRF7. Equivalent amounts of lysates were also used for western blot analysis. All experiments were performed at least three times. Error bars indicate SDs from three independent experiments, ns represents non-significant, * $p < 0.05$, ** $p < 0.01$, *** $p < 0.001$, one-way ANOVA.

Figure S4

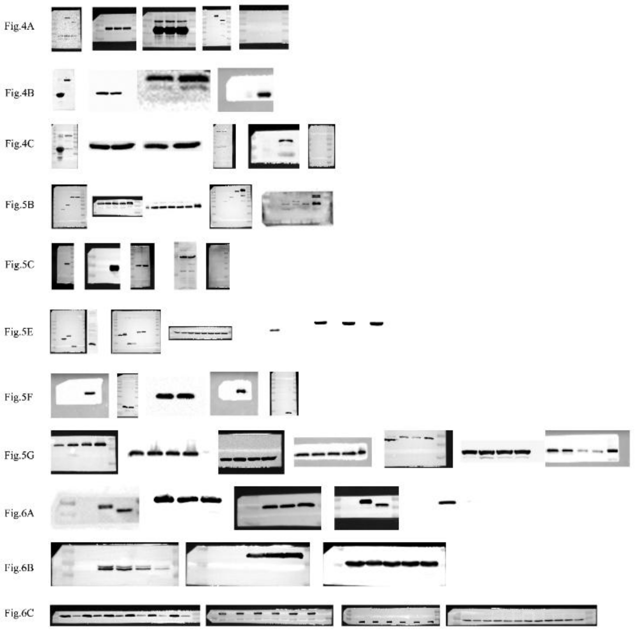

Figure S4. Western blot experiment raw membrane data. Related to Figure 4-6.

Table S1. Sequences of primer.

Gene name	Gene ID	Primers	sequence (5'-3')
KPNA1	NM_002264	Flag-KPNA1-PF	ATGACCACCCCAGGAAAAGAGA
KPNA1	NM_002264	Flag-KPNA1-PR	AAGCTGGAAACCTTCCATAGGA
KPNA2	NM_001320611	Flag-KPNA2-PF	ATGTCCACCAACGAGAATGC
KPNA2	NM_001320611	Flag-KPNA2-PR	AAAGTTAAAGGTCCCAGGAGCC
NuTF2	NM_001322038	Flag-NuTF2-PF	ATGGGAGACAAGCCAATTTGGGA
NuTF2	NM_001322038	Flag-NuTF2-PR	GCCAAAGTTGTGCAGGGCGA
RIG-I	NM_014314	Flag-RIG-I-PF	ATGACCACCGAGCAGCGA
RIG-I	NM_014314	Flag-RIG-I-PR	TCATTTGGACATTTCTGCTGGA
MDA5	NM_022168	Flag-MDA5-PF	ATGTCGAATGGGTATTCCAC
MDA5	NM_022168	Flag-MDA5-PR	CTAATCCTCATCACTAAATAAAC
MAVS	NM_020746	Flag-MAVS-PF	ATGCCGTTTGCTGAAGACAAGA
MAVS	NM_020746	Flag-MAVS-PR	CTAGTGCAGACGCCGC
TBK1	NM_013254	Flag-TBK1-PF	ATGCAGAGCACTTCTAATCATC
TBK1	NM_013254	Flag-TBK1-PR	CTAAAGACAGTCAACGTTG
IRF3	NM_001571	Flag-IRF3-PF	ATGGGAACCCCAAAGCCAC
IRF3	NM_001571	Flag-IRF3-PR	CTAGCTCTCCCCAGGGCCCT
RNF41	NM_001132802	Flag-RNF41-PF	ATGGGGTATGATGTAACCCGTT
RNF41	NM_001132802	Flag-RNF41-PR	CTATATCTCTTCCACACCAT
TBK1-K	N/A	Flag-TBK1-K-PF	ATGCAGAGCACTTCTAATCATCT
TBK1-K	N/A	Flag-TBK1-K-PR	CTAAAAAAACTGGTCAAAACCCCA
TBK1-KU	N/A	Flag-TBK1-KU-PF	ATGCAGAGCACTTCTAATCATCT
TBK1-KU	N/A	Flag-TBK1-KU-PR	CTATACTACAAATATAGGGTT
TBK1-KUS	N/A	Flag-TBK1-KUS-PF	ATGCAGAGCACTTCTAATCATCT
TBK1-KUS	N/A	Flag-TBK1-KUS-PR	CTAATGTTTGATTCCACTGGA
TBK1-US	N/A	Flag-TBK1-US-PF	ATGGCAGAAACTAGTGATATACTTCA
TBK1-US	N/A	Flag-TBK1-US-PR	CTAAAGACAGTCAACGTTG
